# Does the distal screw location in the Biplane double-supported screw fixation technique affect the risk of subtrochanteric fractures after femoral neck fixation? A finite element analysis

**DOI:** 10.1186/s12891-026-09770-5

**Published:** 2026-03-29

**Authors:** Zheng Wang, Xin Wang, Haohui Guo, Bo Bai, Kai Feng, Jianjun Liu, Di Chen, Zhirong Chen

**Affiliations:** 1 The General Hospital of Ningxia Medical University, Ningxia Medical University, Yinchuan, Ningxia China; 2 Orthopedics Ward 3, The General Hospital of Ningxia Medical University, No.804 Shengli South Street, Yinchuan, Ningxia China; 3 Orthopedics Department Trauma Ward 2, The General Hospital of Ningxia Medical University, Yinchuan, Ningxia China

**Keywords:** Femoral neck fracture, BDSF technique, Internal fixation, Pauwels type III femoral neck fracture, Finite element analysis

## Abstract

**Background:**

Research indicates that in the traditional method of cannulated screw fixation for femoral neck fractures, placing the distal screw beyond the lesser trochanter increases the risk of subtrochanteric fractures. In the Biplane double-supported screw fixation technique (BDSF), the lowest screw needs to be inserted at a high inclination angle, making its entry point further from the lesser trochanter. This could increase the risk of iatrogenic subtrochanteric fractures and compromise the stability of the fracture fixation structure. However, there is currently a lack of research on this issue.

**Methods:**

Using BDSF technology to treat Pauwels III femoral neck fractures, we established finite element analysis models with three different distal screw insertion positions (models a, b, c). We determined a range on the lateral cortex midline of the femoral shaft that meets all the requirements for distal screw insertion using BDSF technology. The distal screws were inserted at three positions within this range: upper, middle, and lower, marked as A, B, and C, respectively. Model a: fracture fixation model with insertion point at (A) Model b: fracture fixation model with insertion point at (B) Model c: fracture fixation model with insertion point at (C) Under the same load conditions, finite element analysis was performed on the displacement of the internal fixation and femur, the stress distribution, and the stress distribution in the subtrochanteric region for the three models.

**Results:**

Among the three models we studied, there was no statistically significant difference in the stress magnitude in the subtrochanteric region.Model b had the lowest peak stress values for both the femur and the internal fixation, with displacement values of the femur and internal fixation being close to the minimum. Overall, implanting the distal screw in the middle of the reasonable range of screw placement can provide better stability.

**Conclusions:**

From the perspectives of the maximum displacement of the femur and internal fixation, the maximum stress, and the stress distribution in the subtrochanteric region, implanting the distal screw from the middle position can provide better stability for the fracture ends without increasing the risk of subtrochanteric fractures.

## Introduction

Femoral neck fractures have long posed a critical and complex challenge in orthopedic trauma. Traditionally associated with elderly patients due to osteoporosis, the incidence of these fractures is now increasing in younger populations due to high-energy trauma. Globally, approximately 1.7 million femoral neck fractures occur annually, and this number is projected to reach 6.7 million by 2050 [1]. Selecting an appropriate treatment strategy is essential for restoring patient function and minimizing socioeconomic burden. Among the fracture subtypes, Pauwels type III fractures present particular difficulties due to their steep fracture line and high shear forces, which hinder healing and increase fixation failure risks [2, 3].

For young patients, internal fixation is generally preferred over arthroplasty, not only because of the limited lifespan of prosthetic implants but also due to their higher activity levels, which increase the risk of complications such as implant loosening and periprosthetic fractures [4, 5]. Among various internal fixation strategies, multiple cannulated screws arranged in an inverted triangle configuration remain widely used [6]. This method is minimally invasive and technically simple, providing dynamic compression at the fracture site. However, it often fails to resist shear and torsional forces adequately, resulting in a high incidence of complications such as nonunion, femoral head necrosis, and fixation failure, with reported revision rates as high as 18% [7, 8].

To address the shortcomings of traditional fixation, Filipov introduced the Biplane Double-Supported Screw Fixation (BDSF) technique [9]. This approach employs three partially threaded cannulated screws placed in two distinct planes. The proximal two screws are inserted ventrally and parallel, anchored in thick cortical bone near the femoral shaft to enhance lateral support. The distal screw is positioned dorsally, entering the lateral cortex at a steep angle and contacting the inferior and posterior cortices of the femoral neck, forming an “F” configuration in the anteroposterior view [10]. The BDSF construct significantly improves axial fixation strength (by up to 44%) and has demonstrated a fracture healing rate of 96.6% [11]. Finite element analyses have shown that BDSF effectively neutralizes torsional and shear forces while maintaining axial compressive stress at the fracture site [12].

Nonetheless, concerns remain regarding the potential for iatrogenic subtrochanteric fractures, particularly due to the high-angle insertion of the distal screw, which places the entry point farther from the lesser trochanter. Previous reports have indicated that in inverted triangle constructs, screws positioned distal to the lesser trochanter increase the risk of subtrochanteric fractures [12–14]. These failures are attributed to stress concentration near the calcar region and inadequate posterior cortical support, especially when screw trajectories traverse thin cortical bone in the greater trochanter.

Despite these concerns, there is a lack of biomechanical evidence regarding the influence of distal screw positioning in BDSF constructs. To address this gap, the present study uses finite element analysis to simulate various insertion positions of the distal screw in BDSF fixation. By comparing the stress distribution and displacement patterns in the femur and internal fixation device—as well as evaluating the risk of subtrochanteric fractures—we aim to identify an optimal distal screw trajectory and provide biomechanical support for clinical application of BDSF.

## Materials and methods

### Establishment of the femur model and Pauwels III femoral neck fracture model

**Fig. 1:**
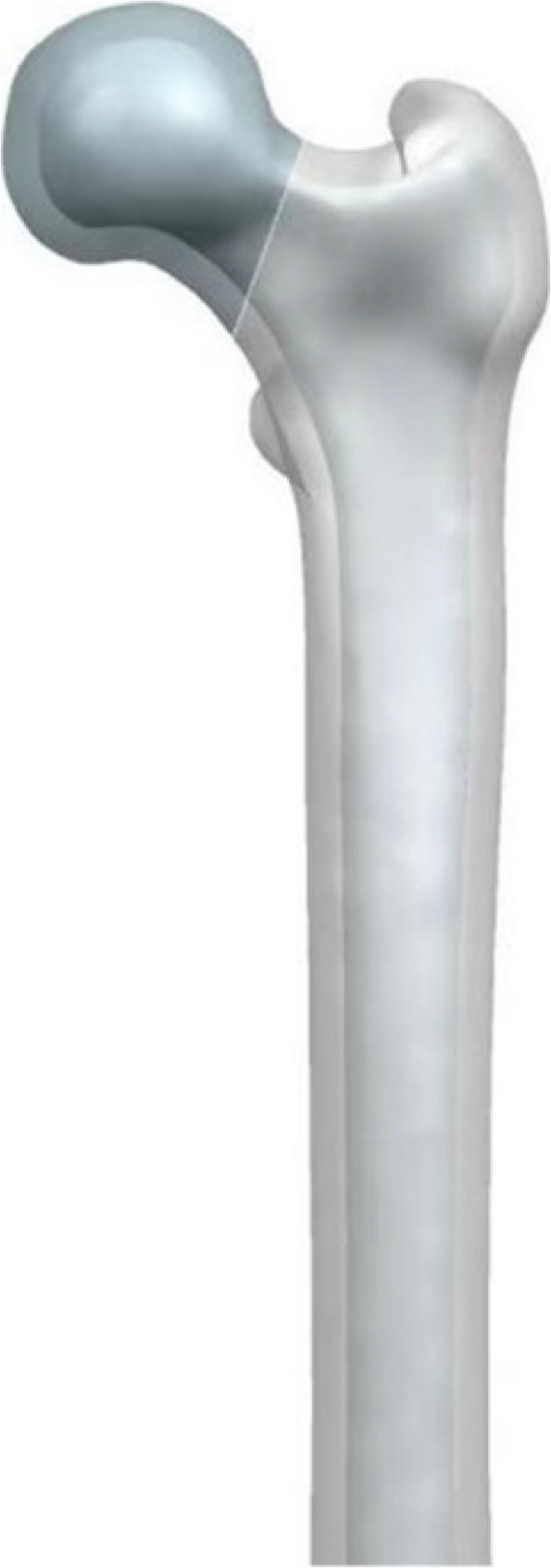
Pauwels III Femoral Neck Fracture Model

The bending and torsional stiffness of composite femurs fall within the range reported for healthy adult femurs under 80 years of age [15], with biomechanical properties comparable to those of young adult femurs. Therefore, a fourth-generation composite left femur (Model 3406; Sawbones, Vashon, WA, USA) was selected as the experimental specimen [16]. This approach avoids ethical concerns associated with using CT data from healthy individuals and minimizes variability.The composite femur was scanned using a 64-slice helical CT scanner (Siemens, Erlangen, Germany) with a slice thickness of 0.625 mm. The CT data were stored in DICOM format and imported into Mimics 21.0 (Materialise, Leuven, Belgium), an interactive medical image processing software. Cortical and cancellous bone were distinguished based on their respective CT values. Using thresholding, region growing, mask editing, contour drawing, and filling, a 3D model of the femur was reconstructed. Basic smoothing operations were applied to improve the surface quality. The resulting STL file was then imported into Geomagic Studio 12.0 (Geomagic, Morrisville, NC, USA) for further refinement. This included repairing discontinuities, filling voids, removing irrelevant structures, and enhancing surface smoothness to obtain a more accurate femur model. To reduce computational load, only the proximal portion of the femur—comprising the femoral head, neck, and upper shaft—was retained. The refined model was exported in IGES format and imported into Siemens NX (NX, Siemens, Germany). On the coronal plane, a perpendicular reference line was drawn through the femoral shaft axis. A cutting plane inclined at 70° to this line was used to simulate a Pauwels type III femoral neck fracture. The fracture model was created by performing a planar cut through the mid-portion of the femoral neck (Fig. 1).

### Establishment of the internal fixation model

According to the clinical data of Synthes screws, we will use NX software to create a parametric model of the cannulated screw. The screw should have a diameter of 7.3 mm, be designed to be self-tapping, and include partial threading.

### Establishment of the surgical model

**Fig. 2:**
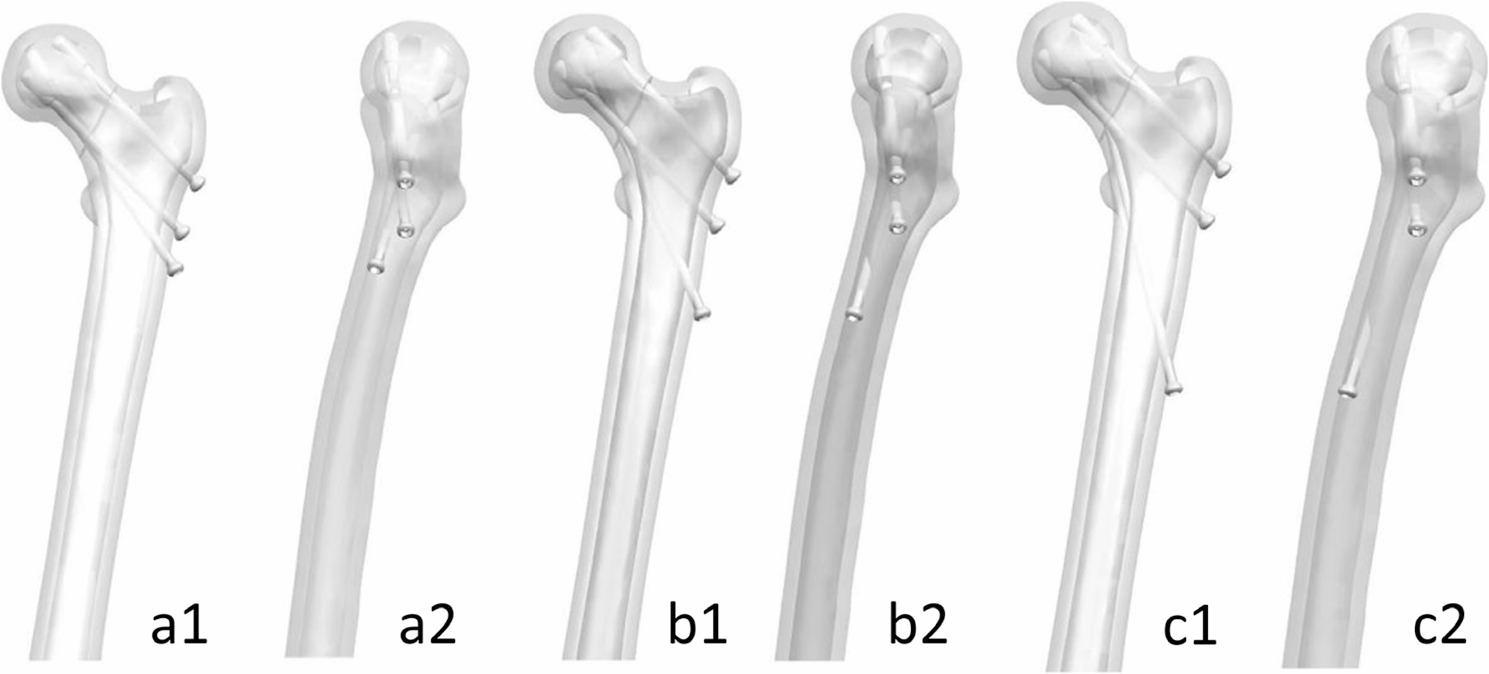
Three Surgical Models of Biplane Double-Supported Screw Fixation Technique with different nail placement positions

The internal fixation implants and Pauwels type III fracture model were imported into Siemens NX software. Using built-in functions such as translation and rotation, the implants were assembled with the fracture model to simulate the surgical process of internal fixation. The Biplane Double-Supported Screw Fixation (BDSF) technique involves three parallel cannulated screws. The placement of the upper two screws followed the principles outlined in the BDSF method [10]. The middle screw was positioned 3–4 cm below the greater trochanter, entering from the posterior one-third of the lateral femoral shaft at an angle of 130°–135° relative to the femoral shaft axis. It passed tangentially to the inferior cortex of the femoral neck and extended into the femoral head. The upper screw was placed 2 cm above the middle screw, parallel to it. The distal screw was implanted last. On the midline of the lateral femoral shaft, all feasible positions satisfying the following conditions were identified: (1) the screw was tangential to the inferior cortex of the femoral neck, (2) tangential to the posterior cortex of the femoral neck, and (3) the screw head was located within the primary compressive trabecular region. Among these, three representative positions—upper, middle, and lower—were selected for screw placement to generate three internally fixed Pauwels type III fracture models. These were labeled models a, b, and c, respectively, according to the order of distal screw position from top to bottom (Fig. 2).

Considering anatomical variability and the need for clinical applicability, we did not adopt a fixed distal screw insertion angle or rely on specific bony landmarks. Instead, we defined a feasible range along the lateral midline of the femoral shaft based on three anatomical criteria:

1. tangency to the inferior cortex of the femoral neck,
2. tangency to the posterior cortex, and.
3. entry point located within the region of primary compressive trabeculae.

**Fig. 3:**
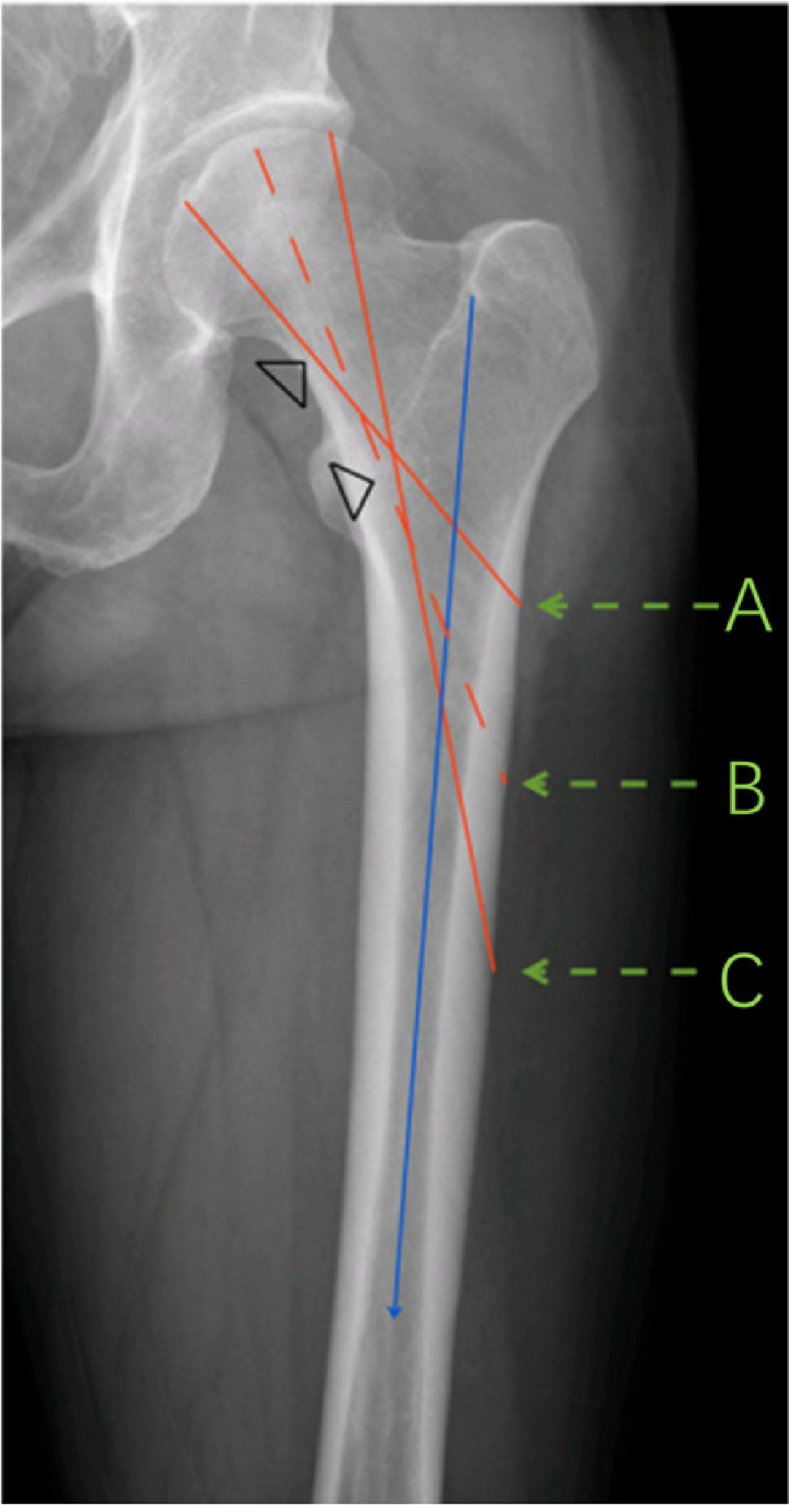
At the femoral head, the region between the red solid lines represents the area of the primary compressive trabeculae. On the lateral side of the femoral shaft, the area between the red solid lines indicates the permissible range for distal screw placement. Arrows A, B, and C denote the three insertion positions from top to bottom

Within this range, we selected three representative positions—upper, middle, and lower—for screw insertion, corresponding to configurations a, b, and c (Fig. 3).

### Model simplification

By simplifying the threaded portion of the screw head to a cylinder with a diameter equal to the outer diameter of the thread, it is possible to better handle the model’s edges, reduce the occurrence of stress singularities, and accurately model the load-bearing points. This simplification approach helps improve the accuracy and stability of the analysis while simplifying the complexity of the model, making the analysis easier to perform and understand, which is a commonly accepted method in finite element analysis.

Although modeling the screws as smooth cylinders is a widely accepted method to reduce computational complexity and avoid stress singularities, it may influence the local stress transfer at the screw-bone interface. In particular, the calcar region, which plays a critical role in mechanical stability, may exhibit different stress patterns in the presence of actual thread geometry. Nevertheless, as all comparative models were subjected to the same simplification, the relative differences in stress distribution and displacement remain valid and unaffected.

### Contact setting

Based on the contact method described in previous literature reports [17, 18], it is assumed that the fracture surface is completely fractured and anatomically reduced. The contact relationship between the two fracture ends has been established, with the fracture surfaces set to have a friction coefficient of 0.46 [19]. Frictional contact is also utilized between the internal fixation device and the bone surface, with a friction coefficient of 0.3 [20]. In all cases, the screw below the BDSF and the bone interface at the femoral shaft are fixed.

### Meshing procedure

The fracture–internal fixation finite element models were meshed using the ANSYS Meshing tool in ANSYS 19.0. All assemblies were meshed with 10-node second-order tetrahedral elements (Solid187). Convergence testing was conducted under different mesh sizes. Using Model a as a representative example, the bony structures were meshed with a 4 mm element size, while the implants were meshed with element sizes of 5 mm, 4 mm, 3 mm, and 2 mm, respectively, using second-order tetrahedral elements. The results showed that when the implant mesh size was refined to 2 mm, the variation in peak Von Mises stress was less than 5%, indicating that convergence had been achieved.Accordingly, all subsequent simulations adopted a 4 mm element size for bone structures and a 2 mm element size for implants, both using 10-node second-order tetrahedral elements. The total number of elements and nodes for the three models are summarized in Table 1.

**Table 1:** The statistics of three assembly elements and the total amount of nodes

Case model	Nodes	Elements
a	277,852	160,965
b	278,260	171,366
c	278,556	171,682

a: The model with the distal screw insertion position at A; b: The model with the distal screw insertion position at B; c: The model with the distal screw insertion position at C.

### Material parameter settings

The various materials in the model are assumed to be homogeneous and isotropic linear elastic materials. The hollow nail is made of a titanium alloy material (Ti-6Al-7Nb) with a Elastic modulus of 110,000 MPa and a Poisson’s ratio of 0.3. The elastic modulus and Poisson’s ratio of cancellous bone are 137 MPa and 0.3, respectively. The elastic modulus and Poisson’s ratio of cortical bone are 16,350 MPa and 0.26, respectively [21]. This information is summarized in Table 2.

**Table 2:** Material propertiesmaterial properties

Material	Elastic modulus (MPa)	Poisson’s ratio
Cortical bone	16,350	0.26
Cancellous bone	137	0.3
Cannulated screw	110,000	0.3

### Boundary conditions and loading force settings

The distal end of the femur was completely constrained with zero degrees of freedom. A loading condition mimicking single-leg stance was applied to simulate the forces exerted on the hip joint during the stance phase of gait [21], To generate bending moments in both the sagittal and coronal planes, as well as rotational moments under axial loading, the femur was internally rotated by 10° and tilted posteriorly by 9° relative to its anatomical position [22]. A static vertical load of 2100 N was applied at the center of the femoral head, corresponding to approximately three times the body weight of a 70 kg adult [23, 24].

### Evaluation criteria

(1) Displacement distribution and peak displacement of femur; (2) Von Mises stress distribution and stress peak value of femur; (3) Von Mises stress distribution and stress peak value of internal fixation; (4) Displacement distribution and peak displacement of the internal fixation; (5) Stress distribution in the subtrochanteric region.

### Statistical analysis

Stress in the subtrochanteric region was analyzed using GraphPad Prism 9.5 (GraphPad Software, Inc.). After confirming homogeneity of variances, one-way ANOVA was performed. A P-value < 0.05 was considered statistically significant.

## Results

### Femoral and Internal Fixation Displacement Peak

**Fig. 4:**
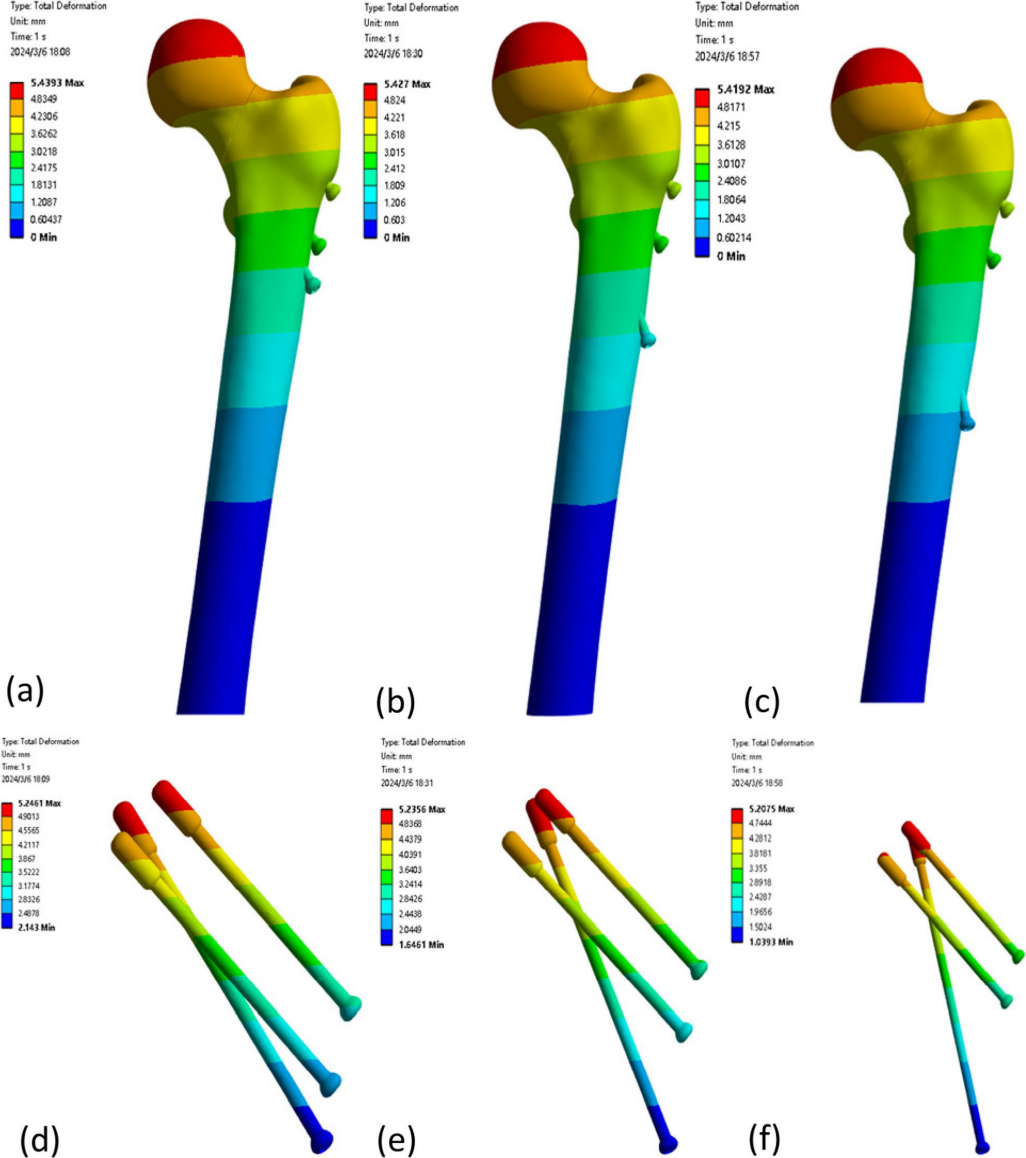
(**a**) Displacement distribution of the femur in Model a; (**b**) Displacement distribution of the femur in Model b; (**c**) Displacement distribution of the femur in Model c; (**d**) Displacement distribution of the internal fixation in Model a; (**e**) Displacement distribution of the internal fixation in Model b; (f) Displacement distribution of the internal fixation in Model c

The maximum femoral displacements of the three models (a: distal screw inserted at position A; b: at position B; c: at position C) were 5.4393 mm, 5.4270 mm, and 5.4192 mm, respectively. The maximum internal fixation displacements were 5.2461 mm, 5.2356 mm, and 5.2075 mm in models a, b, and c, respectively (Fig. 4). Detailed results are presented in Table 3.

**Table 3:** Parameters Results

	a model	b model	c model
The maximum displacement of the femur (mm)	5.4393	5.427	5.4192
The maximum displacement of the Internal fixation (mm)	5.2461	5.2356	5.2075
Maximum femur stress (MPa)	182.47	141.4	149.45
Internal fixation maximum stress (MPa)	131.09	113.46	113.98

### Femoral and internal fixation Von Mises peak stress distribution

**Fig. 5:**
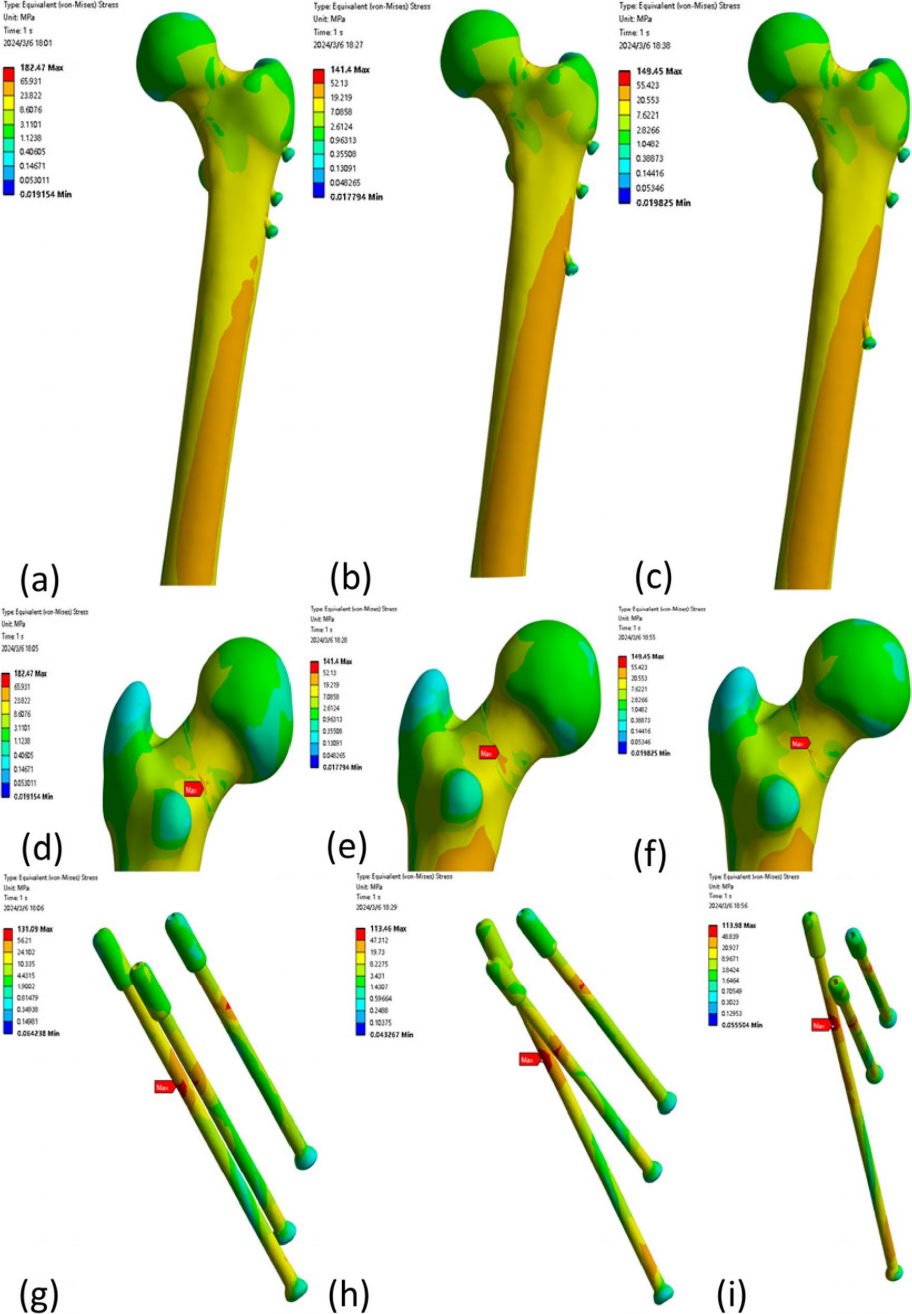
(**a**) Stress distribution of the femur in Model a; (**b**) Stress distribution of the femur in Model b; (**c**) Stress distribution of the femur in Model c; (**d**) Peak stress of the femur in Model a; (**e**) Peak stress of the femur in Model b; (**f**) Peak stress of the femur in Model c; (**g**) Stress distribution and peak stress of the internal fixation in Model a; (**h**) Stress distribution and peak stress of the internal fixation in Model b; (**i**) Stress distribution and peak stress of the internal fixation in Model c

**Fig. 6:**
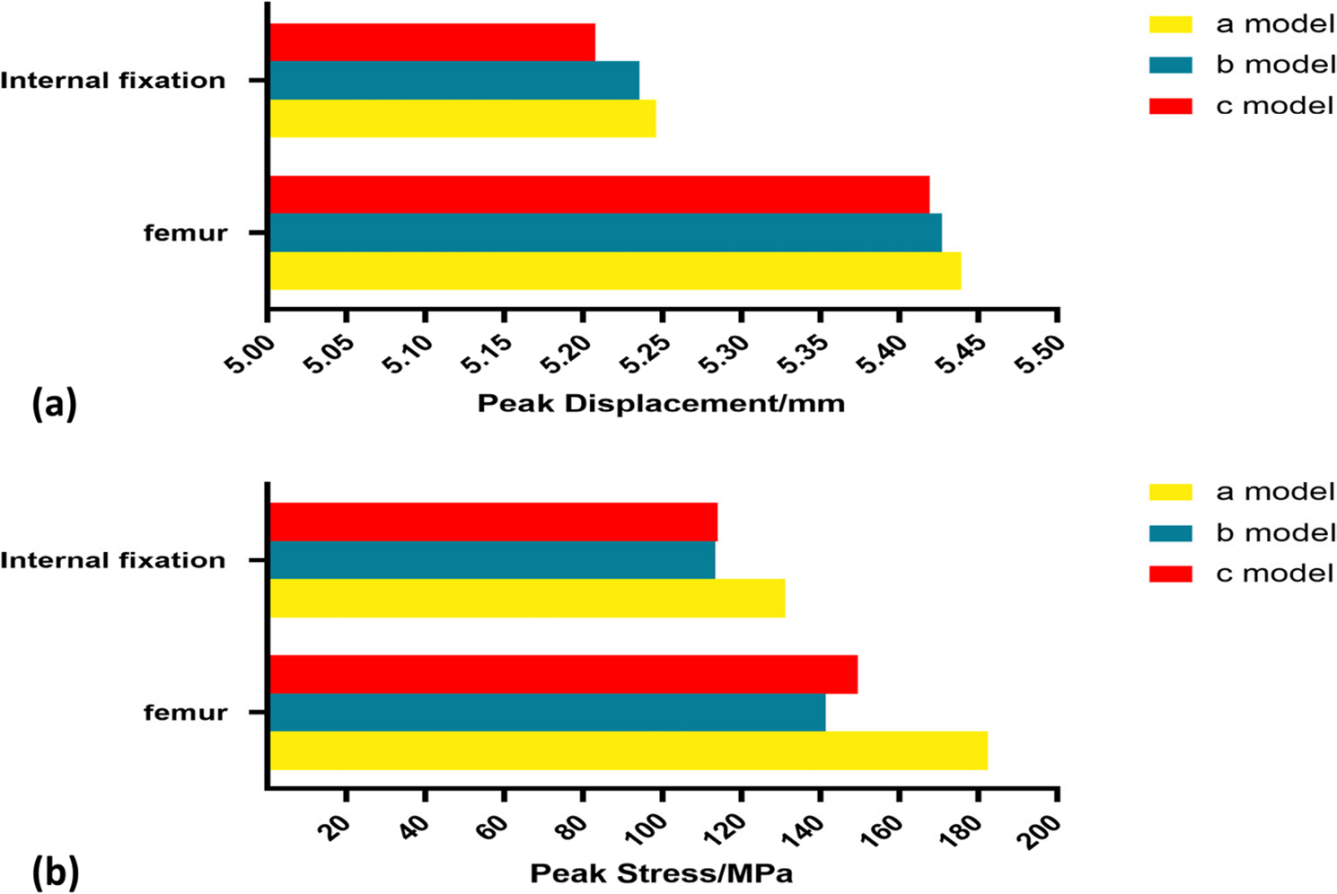
(**a**) Bar graph of peak displacement values of the femur and internal fixation in each model; (**b**) Bar graph of peak stress values of the femur and internal fixation in each model

The peak Von Mises stresses at the femoral head were 182.47 MPa, 141.40 MPa, and 149.45 MPa in models a, b, and c, respectively (Fig. 5). The peak Von Mises stresses of the internal fixation were 131.09 MPa, 113.46 MPa, and 113.98 MPa in models a, b, and c, respectively. Table 2 shows the results in detail. Figure 6 shows the peak histograms of the femur and internal fixation.

### Stress distribution in the subtrochanteric region

**Fig. 7:**
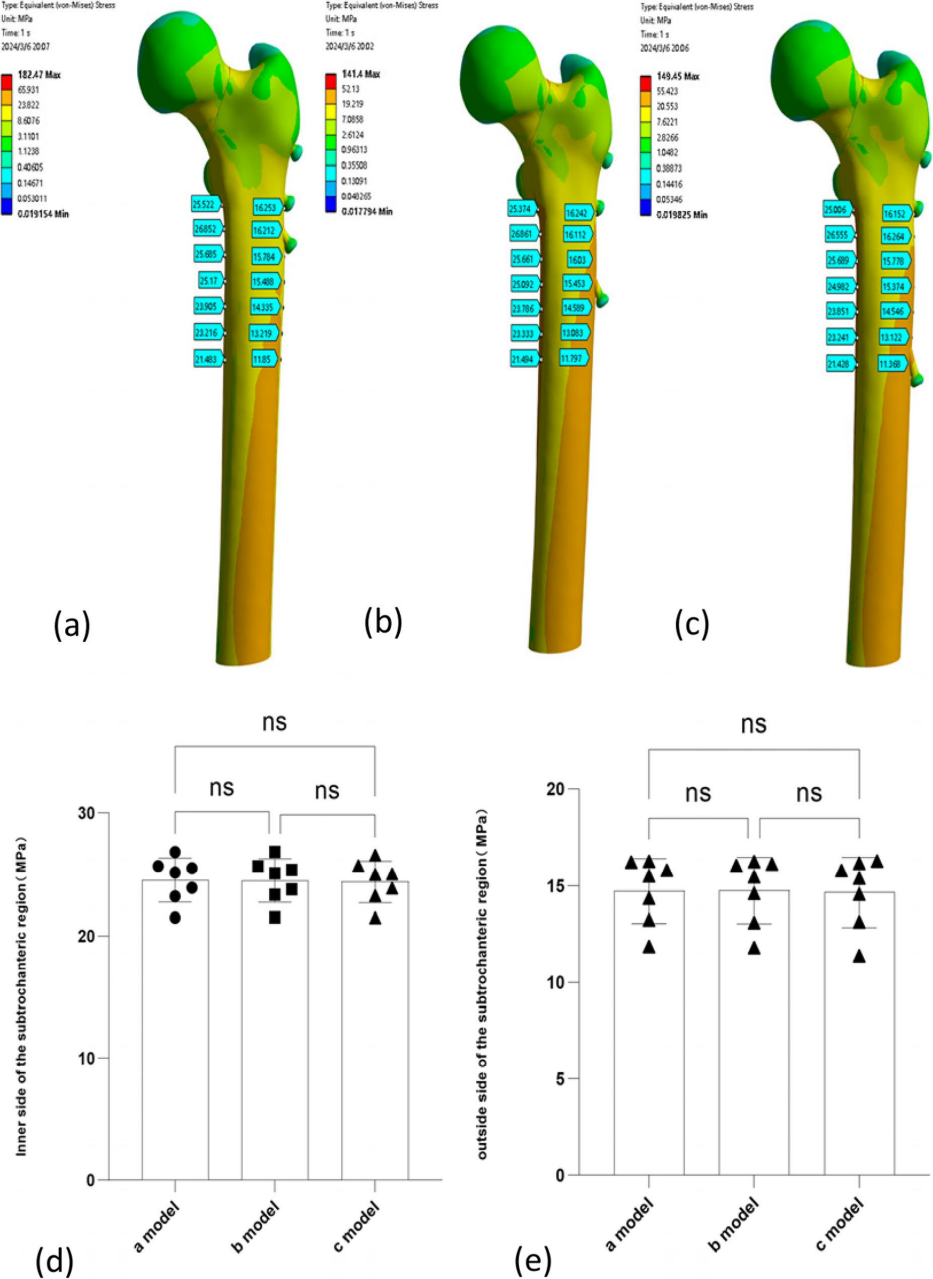
(**a**) Medial and lateral stress distribution in the subtrochanteric region of the femur in Model a; (**b**) Medial and lateral stress distribution in the subtrochanteric region of the femur in Model b; (**c**) Medial and lateral stress distribution in the subtrochanteric region of the femur in Model c; (**d**) Bar graph of medial stress in the subtrochanteric region across the three models, with no statistically significant differences observed; (**e**) Bar graph of lateral stress in the subtrochanteric region across the three models, with no statistically significant differences observed. One-way ANOVA were used to compare the data, *p* > 0.05

A 7 cm segment of the femur, starting below the lesser trochanter, was selected and evenly divided into seven sections. At each section, the stress on both the medial and lateral sides of the femur was calculated. The stress distributions in the subtrochanteric region for models a, b, and c are shown in Fig. 7 and summarized in Table 4.

**Table 4:** Parameters Results

		1	2	3	4	5	6	7
Stress with Inner side of the subtrochanteric region (MPa)	a model	25.522	26.852	25.685	25.17	23.905	23.216	21.483
	b model	25.374	26.861	25.661	25.092	23.786	23.333	21.494
	c model	25.006	26.555	25.689	24.982	23.851	23.241	21.428
Stress with outside side of the subtrochanteric region (MPa)	a model	16.253	16.212	15.784	15.488	14.335	13.219	11.85
	b model	16.242	16.112	16.03	15.453	14.589	13.083	11.797
	c model	16.152	16.264	15.778	15.374	14.546	13.122	11.368

## Discussion

### Mechanical insights

This study systematically evaluated the biomechanical effects of varying distal screw positions in the BDSF technique for femoral neck fractures using finite element analysis. The results indicate that screw placement plays a critical role in stress distribution and overall construct stability. In all three models, peak stress was concentrated in the posterior cortical region of the femoral neck rather than at the calcar femorale, consistent with prior reports on BDSF biomechanics [12, 25]. Notably, Model a exhibited a peak stress of 182.47 MPa, approaching the upper limit of the femoral ultimate load-bearing capacity in healthy adults (170–190 MPa) [26, 27], suggesting an elevated biomechanical risk associated with high-position screw placement.In contrast, Models b and c, which featured more inferior placement of the distal screw, effectively reduced calcar stress via a cantilever beam–like effect. However, their screw trajectories differed. In Model b, the distal screw intersected the midsection of the fracture line at a near-perpendicular angle, with the head located in the superomedial femoral head—providing superior resistance to posterior torsional stress. In Model c, the screw was positioned more posteriorly and vertically, partially compromising its resistance to torsional forces, it is also the reason for the subtle gap between the two.Although the stress levels in Models b and c (141 MPa) were below the osteoporotic fracture threshold of 150 MPa [28], caution is advised, as transient impact loads during daily activities (e.g., falls) may reach two to three times the static values.

Additionally, the stress distribution within the screws followed a consistent trend across all models: stress decreased from the midsection of the screw toward both ends. Although the original mechanical concept of the BDSF technique posits that a lower screw placement increases the lever arm and reduces stress at the fulcrum (calcar femorale), our study revealed a dynamic shift in the fulcrum location depending on screw position. This resulted in less reduction of calcar stress than theoretically expected in Models c, thereby challenging the conventional assumption that “the lower, the better” for distal screw placement. This nuanced insight highlights the need for individualized screw positioning rather than a one-size-fits-all approach.

### Position-dependent performance of the BDSF technique

Compared with the conventional inverted triangle fixation technique—whose reported peak stress under similar finite element conditions was 182.40 MPa [25]—our results emphasize that the biomechanical advantages of the BDSF approach are clearly position-dependent: 1) The BDSF technique effectively alleviates stress concentration at the calcar femorale, validating findings from prior studies [12, 25]. 2) The low-position screw configurations (Models b and c), through a cantilever beam design, demonstrated mechanical advantages that align with Filipov et al.’s theoretical model. However, our results further indicate that the magnitude of this advantage is modulated by patient-specific anatomical variation [29]. 3) With respect to subtrochanteric stress, no statistically significant differences were observed among the models (*P* > 0.05). This finding contrasts with results from conventional fixation techniques and supports the overall mechanical safety of BDSF regardless of screw level in that specific region [13]. Although our finite element analysis indicates that distal placement of the screw does not necessarily increase stress concentration in the subtrochanteric region, this conclusion is based on a simplified model that does not account for all biological and mechanical variables present in vivo. Contradictions with previous clinical findings suggest that further investigation through prospective or retrospective clinical studies is needed to validate the biomechanical predictions presented here.

### Clinical relevance

The biomechanical results from this study provide practical guidance for surgical decision-making. Different distal screw positions offer distinct advantages and trade-offs that should be carefully considered in the clinical setting. 1) High-position screw (Model a): This configuration is technically easier to implement and may therefore be favored by less experienced surgeons. Although it generated elevated posterior femoral neck stress in the finite element analysis, the direct clinical translation of this finding into an increased refracture risk should be interpreted with caution, as such events are rare in the presence of internal fixation. Nevertheless, in patients with poor bone quality, particularly those with osteoporosis, thorough preoperative assessment is warranted, and supplemental fixation or alternative screw positioning may be considered. 2) Mid-position screw (Model b): Based on mechanical properties and surgical feasibility, the mid-portion of the feasible screw trajectory appears to be the optimal insertion zone. This configuration requires greater technical skill and may be selected by more experienced surgeons aiming to maximize biomechanical stability. It strikes a balance between stability and safety and can be considered the first-choice strategy for most patients. It is particularly beneficial for those with osteoporosis (T-score < − 2.5), vertically oriented fracture lines (Pauwels angle > 50°), or long life expectancy requiring durable fixation. 3) Low-position screw (Model c): Although this configuration demonstrated superior mechanical performance, it requires longer implants and poses additional clinical challenges, including increased surgical complexity (e.g., avoiding repeated guidewire placement), a higher risk of vascular injury, and elevated procedural costs.

To ensure safe intraoperative application, guidewire placement should be confirmed under C-arm/G-arm fluoroscopy within no more than three attempts to minimize repeated puncture and avoid potential compromise of the femoral head blood supply. Postoperative CT scans at 6 weeks may also be valuable for detecting early healing patterns and identifying stress concentration zones, such as microfractures in the posterior femoral neck cortex.Taken together, these findings highlight that screw positioning in the BDSF technique should be individualized according to both patient-specific factors (e.g., bone quality, fracture morphology) and surgeon experience. This decision-making framework, which integrates finite element biomechanics with clinical feasibility, represents an important step toward individualized surgical planning. Future validation through multicenter clinical trials is warranted to confirm its clinical utility.

## Limitations and future directions

Despite the valuable insights provided by this study, several limitations must be acknowledged.First, the finite element models were based on a single artificial femur and did not account for individual variability in bone mineral density or geometry. The periarticular soft tissues were simplified, and the loading conditions were idealized, which may not fully represent in vivo biomechanics. Additionally, the threaded portions of the screws were modeled as smooth cylinders to avoid stress singularities and reduce computational complexity. While this approach is widely used, it may affect local stress transfer, particularly in the calcar region where thread engagement plays a crucial role.Second, the study design involved a limited sample size (*n* = 3), lacked fatigue testing under cyclic loading, and did not incorporate multi-parametric or patient-specific coupling analyses.Third, screw positions (Models a–c) were defined relative to a feasible insertion range rather than fixed bony landmarks. Although this method accommodates anatomical variability, it may reduce intraoperative reproducibility. Future studies incorporating measurements from landmarks such as the greater trochanter (LT) could improve surgical applicability and standardization.

To address these limitations, future research should proceed along two complementary directions. From a basic research perspective, it is essential to develop a comprehensive finite element model library that incorporates bone density gradients, anatomical variability, and more realistic soft tissue structures. Validation through cadaveric biomechanical testing will further strengthen the credibility of the computational findings. From a clinical standpoint, multicenter randomized controlled trials (RCTs) should be conducted to evaluate long-term outcomes. Moreover, integrating artificial intelligence into preoperative planning, establishing standardized surgical protocols, and developing patient-specific surgical guides tailored to the BDSF technique will facilitate clinical translation and broader application.

## Conclusion

This study systematically investigated the biomechanical effects of distal screw positioning in the Biplane Double-Supported Screw Fixation (BDSF) technique using finite element analysis. The following key conclusions were drawn:1)The relationship between screw position and biomechanical performance is nonlinear, indicating that the conventional view of “the lower, the better” requires reconsideration.2༉Mid-position screw placement offers the optimal balance between mechanical stability and surgical feasibility.3༉Low-position screw configurations do not increase the risk of subtrochanteric fracture; however, they demand higher surgical precision and technical skill.4༉Preoperative planning protocols should be developed based on patient-specific anatomical and biomechanical characteristics.These findings not only refine the theoretical foundation of the BDSF technique but also provide a scientific basis for its standardized clinical application. Future studies should focus on clinical validation and the development of standardized operative guidelines for broader implementation.

## Data Availability

The datasets used and/or analyzed during the current study are available from the corresponding author on reasonable request.

## Abbreviations

BDSF: Biplane double-supported screw fixation
CT: Computed tomography
NX: Siemens NX
ANOVA: Analysis of variance

## References

[1] Miyamoto RG, Kaplan KM, Levine BR, Egol KA, Zuckerman JD. Surgical management of hip fractures: an evidence-based review of the literature. I: femoral neck fractures. JAAOS. 2008;16(10):596–607. 18832603 10.5435/00124635-200810000-00005

[2] Davidovitch RI, Jordan CJ, Egol KA, Vrahas MS. Challenges in the treatment of femoral neck fractures in the nonelderly adult. J Trauma. 2010;68(1):236–42. 20065780 10.1097/TA.0b013e3181c428ce

[3] Estrada LS, Volgas DA, Stannard JP, Alonso JE. Fixation failure in femoral neck fractures. Clin Orthop Relat Res. 2002(399):110–8. 10.1097/00003086-200206000-00013 12011699

[4] Panteli M, Rodham P, Giannoudis PV. Biomechanical rationale for implant choices in femoral neck fracture fixation in the non-elderly. Injury. 2015;46(3):445–52. 25597514 10.1016/j.injury.2014.12.031

[5] Bhandari M, Devereaux PJ, Swiontkowski MF, Tornetta P, Obremskey W, Koval KJ, Nork S, Sprague S, Schemitsch EH, Guyatt GH. Internal fixation compared with arthroplasty for displaced fractures of the femoral neck. A meta-analysis. J Bone Joint Surg Am. 2003;85(9):1673–81. 12954824 10.2106/00004623-200309000-00004

[6] Duffin M, Pilson HT. Technologies for Young Femoral Neck Fracture Fixation. J Orthop Trauma. 2019;33(Suppl 1):S20–6. 30540668 10.1097/BOT.0000000000001367

[7] Augat P, Bliven E, Hackl S. Biomechanics of Femoral Neck Fractures and Implications for Fixation. J Orthop Trauma. 2019;33(Suppl 1):S27–32. 30540669 10.1097/BOT.0000000000001365

[8] Slobogean GP, Sprague SA, Scott T, Bhandari M. Complications following young femoral neck fractures. Injury. 2015;46(3):484–91. 25480307 10.1016/j.injury.2014.10.010

[9] Filipov O. Biplane double-supported screw fixation (F-technique): a method of screw fixation at osteoporotic fractures of the femoral neck. Eur J Orthop Surg Traumatol. 2011;21(7):539–43. 21966288 10.1007/s00590-010-0747-9 PMC3178023

[10] Filipov OB. Biplane Double-supported Screw Fixation of Femoral Neck Fractures: Surgical Technique and Surgical Notes. JAAOS. 2019;27(11):e507–15. 30399029 10.5435/JAAOS-D-17-00117 PMC6530978

[11] Filipov O, Stoffel K, Gueorguiev B, Sommer C: Biomechanics and indications for application of the method of BDSF. Answer to manuscript draft number AOTS-D- 17–00378, Letter to the concerning ‘’Femoral neck fracture osteosynthesis by the biplane double-supported screw fixation method (BDSF) reduces the risk of fixation failure: clinical outcomes in 207 patients’’ by, Filipov O, Sommer C et al. (2017) Arch Orthop Trauma Surg. Apr 8. [Epub ahead of print]. Arch Orthop Trauma Surg 2017;137(8):1167–1171. 10.1007/s00402-017-2716-9 PMC5511324 28667396

[12] Tianye L, Peng Y, Jingli X, QiuShi W, GuangQuan Z, Wei H, Qingwen Z. Finite element analysis of different internal fixation methods for the treatment of Pauwels type III femoral neck fracture. Biomed Pharmacother. 2019;112:108658. 30970508 10.1016/j.biopha.2019.108658

[13] Crump EK, Quacinella M, Deafenbaugh BK. Does Screw Location Affect the Risk of Subtrochanteric Femur Fracture After Femoral Neck Fixation? A Biomechanical Study. Clin Orthop Relat Res. 2020;478(4):770–6. 32229749 10.1097/CORR.0000000000000945 PMC7282603

[14] Hickey B, Jones HM, Jones S. Is distal screw entry point associated with subtrochanteric fracture after intracapsular hip fracture fixation? ANZ J Surg. 2014;84(4):245–8. 24812708 10.1111/ans.12498

[15] Gardner MP, Chong ACM, Pollock AG, Wooley PH. Mechanical evaluation of large-size fourth-generation composite femur and tibia models. Ann Biomed Eng. 2010;38(3):613–20. 20049637 10.1007/s10439-009-9887-7

[16] Heiner AD. Structural properties of fourth-generation composite femurs and tibias. J Biomech. 2008;41(15):3282–4. 18829031 10.1016/j.jbiomech.2008.08.013

[17] Chen W-P, Tai C-L, Shih C-H, Hsieh P-H, Leou M-C, Lee MS. Selection of fixation devices in proximal femur rotational osteotomy: clinical complications and finite element analysis. Clin Biomech (Bristol Avon). 2004;19(3):255–62. 10.1016/j.clinbiomech.2003.12.003 15003340

[18] Samsami S, Saberi S, Sadighi S, Rouhi G. Comparison of Three Fixation Methods for Femoral Neck Fracture in Young Adults: Experimental and Numerical Investigations. J Med Biol Eng. 2015;35(5):566–79. 26500470 10.1007/s40846-015-0085-9 PMC4609309

[19] Hunt SA, Martin RH, Woolridge B. Fatigue Testing of a New Locking Plate for Hip Fractures. J Med Biol Eng. 2012;32:117–22.

[20] Eberle S, Gerber C, von Oldenburg G, Högel F, Augat P. A biomechanical evaluation of orthopaedic implants for hip fractures by finite element analysis and in-vitro tests. Proc Inst Mech Eng H. 2010;224(10):1141–52. 21138232 10.1243/09544119JEIM799

[21] Zhang B, Liu J, Zhang W. Ordinary Cannulated Compression Screws or Headless Cannulated Compression Screws? A Synthetic Bone Biomechanical Research in the Internal Fixation of Vertical Femoral Neck Fracture. Biomed Res Int. 2018;2018:4898301. 29850523 10.1155/2018/4898301 PMC5925079

[22] Li J, Yin P, Zhang L, Chen H, Tang P. Medial anatomical buttress plate in treating displaced femoral neck fracture a finite element analysis. Injury. 2019;50(11):1895–900. 31455504 10.1016/j.injury.2019.08.024

[23] Zhang Y, Tian L, Yan Y, Sang H, Ma Z, Jie Q, Lei W, Wu Z. Biomechanical evaluation of the expansive cannulated screw for fixation of femoral neck fractures. Injury. 2011;42(11):1372–6. 21824615 10.1016/j.injury.2011.07.004

[24] Madsen F, Linde F, Andersen E, Birke H, Hvass I, Poulsen TD. Fixation of displaced femoral neck fractures. A comparison between sliding screw plate and four cancellous bone screws. Acta Orthop Scand. 1987;58(3):212–6. 3307282 10.3109/17453678709146468

[25] Lin S, Shang J, Xing B, Wu B, Peng R, Wang G, Lu H-D. Modified F configuration in the treatment of Pauwels type III femoral neck fracture: a finite element analysis. BMC Musculoskelet Disord. 2021;22(1):758. 34488708 10.1186/s12891-021-04638-2 PMC8420054

[26] Bayraktar HH, Morgan EF, Niebur GL, Morris GE, Wong EK, Keaveny TM. Comparison of the elastic and yield properties of human femoral trabecular and cortical bone tissue. J Biomech. 2004;37(1):27–35. 14672565 10.1016/s0021-9290(03)00257-4

[27] Schileo E, Taddei F, Malandrino A, Cristofolini L, Viceconti M. Subject-specific finite element models can accurately predict strain levels in long bones. J Biomech. 2007;40(13):2982–9. 17434172 10.1016/j.jbiomech.2007.02.010

[28] Bevill G, Keaveny TM. Trabecular bone strength predictions using finite element analysis of micro-scale images at limited spatial resolution. Bone. 2009;44(4):579–84. 19135184 10.1016/j.bone.2008.11.020

[29] Filipov O, Stoffel K, Gueorguiev B, Sommer C. Femoral neck fracture osteosynthesis by the biplane double-supported screw fixation method (BDSF) reduces the risk of fixation failure: clinical outcomes in 207 patients. Arch Orthop Trauma Surg. 2017;137(6):779–88. 28391429 10.1007/s00402-017-2689-8 PMC5432592

